# O_2_ Dissociative Adsorption on Mg(0001)—Surface Oxidation, Peroxide Formation, and Oxide Layer Thickening

**DOI:** 10.1021/acsomega.5c02179

**Published:** 2025-06-02

**Authors:** Yunyan Han, Haijun Jiao

**Affiliations:** † Key Laboratory of Eco-Functional Polymer Materials of the Ministry of Education, College of Chemistry and Chemical Engineering, 12435 Northwest Normal University, Lanzhou 730070, China; ‡ 28392 Leibniz-Institut für Katalyse e.V. (LIKAT), Albert-Einstein-Str. 29A, Rostock 18059, Germany

## Abstract

O_2_ exposure-dependent oxidation process on the *p*(4 × 4) Mg(0001) surface has been systematically studied based on DFT computation and AIMD analysis. At initial exposure, O_2_ dissociates spontaneously, and the oxygen atoms penetrate the subsurface layers, forming oxide islands due to the electrostatic attractive Mg–O interaction. The oxide islands grow laterally and vertically with an increase in the level of exposure to O_2_ and eventually, the first two stoichiometrically oxidized layers (32 × O) are formed. The oxidized surface layer can further uptake both oxygen atoms and molecular O_2_ and forms stable mixed adsorption configurations (4O+xO_2_, x = 1–4), which reveal the formation of surface peroxides, as proposed by an XPS study. The AIMD analysis explains the experimentally observed changes in the LEED patterns during the oxidation process upon the increase of O_2_ exposure.

## Graphical abstract

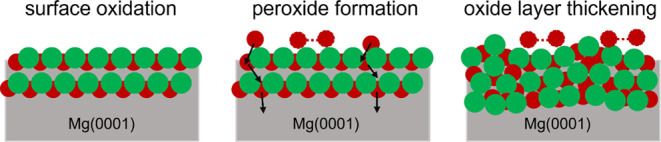

## Introduction

With growing pressure from climate change and energy crises, lightweight technology as an effective way to reduce fuel consumption and CO_2_ emission as well as energy cost is becoming increasingly important.^1^ As the lightest structural metal, magnesium has been widely applied in various fields such as transportation, energy storage, electronics, and biomedical technologies due to its unique properties, including high specific strength and stiffness, high thermal and electrical conductivity, and biocompatibility.^2–4^ However, magnesium and its alloys are highly active and corrosive, causing material damage and enormous loss for the economy, which severely limit their further development.^1,5,6^ To address this problem, a good understanding of the corrosion mechanisms is essential.^7^ Upon being exposed to air, magnesium surfaces are easily oxidized, and the oxidized layers are often considered as the first line of defense against corrosion, especially under atmospheric conditions. Thus, it is important to gain deep insight into the oxidation process, as well as the structure, composition, thickness, and properties of the oxidized layers, for the corrosion protection of magnesium.^8,9^

The surface oxidation of poly crystalline^10–14^ and single crystals^15–20^ of magnesium has been well investigated by many experimental techniques, including X-ray photoelectron spectroscopy (XPS),^10–13^ ultraviolet photoelectron spectroscopy (UPS),^11,12^ synchrotron radiation photoelectron spectroscopy (SRPS),^14^ low-energy electron diffraction (LEED),^15–17,19,20^ Auger electron spectroscopy (AES),^12,16,19^ electron energy-loss spectroscopy (ELS),^15–17,19^ scanning tunneling microscopy (STM),^20^ ellipsometry,^17,18^ and work function measurements.^16,17^ It is generally accepted that the oxidation involves three main steps: oxygen incorporation, monolayer completion, and film thickening. Under initial exposure to O_2_, O_2_ dissociates on the magnesium surface and then penetrates the subsurface region to form oxide nuclei. Upon further exposure to O_2_, the incorporated oxygen atoms aggregate into islands, which grow rapidly and epitaxially until the formation of two or three monolayer oxide islands. With a further increased exposure to O_2_, the oxide film thickens slowly. It has been reported that the thickness of the oxide layer after saturation exposure was estimated to be 7 ± 3 Å for polycrystalline magnesium^12^ and about 10 Å for the Mg(0001) surface.^16^ Under water vapor and humid air, Chen et al.^21,22^ found a similar three-step oxidation process in the growth of the oxide film. By using STM, Goonewardene et al.^20^ carefully tracked the changes in the Mg(0001) surface under O_2_ and monitored the structural evolution of the oxide layer from initial pillars to bumps with a height growth from 1.2 to 3.6 Å, followed by area growth, and finally a MgO(111)-like close-packed structure.

Although most experiments are consistent with the three-step process during magnesium oxidation, the nature of the incorporated oxygen atoms is still debated. For example, the assignment of the XPS signal at ∼533 eV associated with incorporated oxygen atoms still remains controversial. It appears only after the development of the main signal with low binding energy at ∼531 eV assigned to magnesium oxide at high exposure, and its intensity decreases with an increase in temperature. Barteau^8^ proposed that this signal may originate from the hydroxyl groups due to the residual H_2_O in the chamber on the MgO surface, while Ghijsen et al.^23^ assigned this signal to oxygen atoms incorporated within the already oxidized magnesium area in the substrate (defect oxide). By studying the oxidation of ultrathin films of magnesium supported on Mo(100) using XPS in the 90–1300 K range, Corneille et al.^24^ concluded that the ∼533 eV signal arises from the existence of a peroxide state. Under soft X-ray synchrotron radiation, Malik et al.^25^ monitored the valence band spectra of magnesium thin films exposed to O_2_ on Ru(001) and found several new signals at high O_2_ exposure, which could be attributed to the presence of molecular dioxygen species, most probably MgO_2_.

In addition to the extensive experimental studies, theoretical analysis on the oxidation of magnesium surfaces has also been carried out.^9,26–30^ DFT (GGA-RPBE) calculations by Hellman^9^ showed that there is no energy barrier for O_2_ dissociation. Bungaro et al.^26^ studied the early oxidation stage of Mg(0001) using DFT (LDA) and found that oxygen is adsorbed below the Mg surface, forming ionic islands commensurate with the metal lattice, in agreement with experiments. Schröder et al.^27–29^ computed both early and intermediate oxidation states of the Mg(0001) surface using DFT (GGA) and found that O is incorporated below the topmost Mg layer in tetrahedral sites at very low coverage, and O is adsorbed onto on-surface or subsurface sites to form dense clusters with the increase of coverage; this leads to the formation of a thin layered and directionally bound surface oxide on top of an almost unchanged Mg(0001) surface. Francis et al.^30^ computed the binding of oxygen to Mg(0001) and subsequent clustering using DFT­(GGA-PW91) and concluded that magnesium mediates an attractive oxygen–oxygen interaction that ultimately leads to the formation of hexagonal clusters of O* at the tetrahedral-1 site.

Despite these experimental and DFT studies, several problems remain unsolved, such as the changes in the structures of magnesium surfaces (including the clustering process and film thickening) with increasing oxygen exposure, and the corresponding thermodynamics and kinetics. Additionally, the nature of adsorbed oxygen atoms during oxidation remains unclear. Based on this background, we applied periodic DFT computations and ab initio molecular dynamics (AIMD) simulations to study in detail the exposure-dependent thermodynamics and kinetics of the dissociative adsorption of molecular oxygen (O_2_), the diffusion of the incorporated oxygen atoms, and the nature of differently located oxygen atoms, especially the formation, stability, and reactivity of surface peroxides and thickening of the oxide layers. This study provides a more comprehensive understanding of the oxidation process of Mg at the atomic level, which should be useful for the corrosion protection of magnesium surfaces.

## Computation Models and Methods

All calculations were performed based on the periodic slab model using the plane-wave-based DFT method implemented in the Vienna Ab initio Simulation Package (VASP).^31–33^ The projected augmented wave method (PAW)^34,35^ was used to describe the interaction of electron and ion. The electron exchange and correlation energies were calculated within the generalized gradient approximation method (GGA) using the Perdew–Burke–Ernzerhof (PBE) functional.^36^ All simulations were performed using a 2 × 2 × 1 gamma-centered grid of k-points. The plane-wave expansion was limited by a cutoff energy of 520 eV. Structure optimization was converged until the forces acting on the atoms were smaller than 0.03 eV/Å, whereas the energy threshold-defining self-consistency of the electron density was set to 10^– 5^ eV. For bulk calculations, the cell parameters were relaxed (ISIF = 3). And for the slab calculations, the cell parameters were fixed (ISIF = 2). The PBE-D3 method was employed in all DFT computations for the dispersion correction.^37^ The climbing image nudged elastic band (CI-NEB) method^38,39^ was used to find the transition state, and four images were used in the band. Frequency analysis was carried out to characterize authentic transition states with only one imaginary frequency. Zero-point energy (ZPE) correction was included in the energy calculations.

The error between the calculated lattice parameters of the hexagonal close-packed structure (c = 5.11, a = 3.15 Å) and the experimental values (c = 5.21, a = 3.21 Å)^40^ are less than 2.0%. The computed interlayer relaxation also agrees with the measurements from LEED at 130 K^41^ (Table S1), and both capture the unusual outward relaxation of the clean Mg(0001) surface. The computed surface energy of the clean magnesium surface agrees with the experimental value (0.81 J/m^2^ vs 0.78 J/m^2^).^42^

A six-layer slab model with the bottom three-layer frozen and the upper three-layer along with adsorbates relaxed freely was used, in which a 15 Å vacuum gap was created between the uppermost and bottom-most layers. And a p (4 × 4) model of Mg(0001) was used (64 Mg atoms in total). The top and side views of the model are shown in Figure 1, and the top three layers allowed to relax are clearly distinguished by color from light to dark. The surface layer contains face-centered cubic (fcc), hexagonal-closed packed (hcp), top and bridge (bri) sites for adsorption. The first sublayer includes a tetrahedral site under the top site (TUT1), a tetrahedral site under the hcp site (TUH1), and an octahedral site (Oct1). The second sublayer features a tetrahedral site under the top site (TUT2), a tetrahedral site under the hcp site (TUH2), and an octahedral site (Oct2).

**Figure 1:**
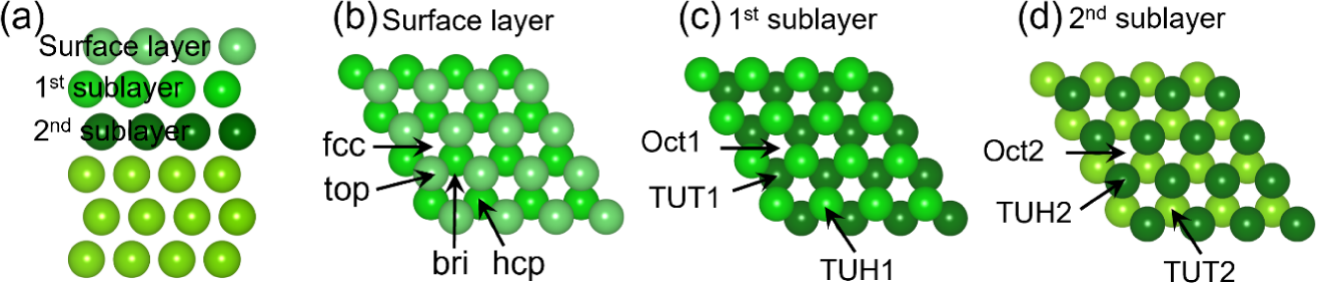
(a) Side view of the surface layer, the first sublayer, and the second layer; (b) top view and adsorption sites of the surface layer; (c) top view and adsorption sites of the first sublayer (by removal of the surface layer); (d) top view and adsorption sites of the second sublayer (by removal of the first two layers) of the p(4 × 4) Mg(0001) slab model.

In addition to the DFT calculations, AIMD simulations of the collision of the O_2_ molecules with the Mg(0001) surface and the subsequent adsorption and dissociation were performed at 300 K to acquire a more detailed understanding of the oxidation process. Initially, AIMD simulations were conducted using the fully relaxed slab model in a constant pressure ensemble (NPT) at 300 K and 1 atm pressure for a duration of 10 picoseconds (ps) to allow the adjustment of the simulation cell axes, ensuring isotropic stress across the slab system. Then, the oxidation simulations were performed at 300 K in a canonical (NVT) ensemble with a timestep of 1 femtosecond (fs), where the system cell axes were fixed at the values obtained from the NPT simulations, and the bottom two layers were fixed. To simulate the ultrahigh vacuum experiment of Mg(0001) surface oxidation and gain a better view of the oxidation process, O_2_ molecules were added to the gas phase one by one onto the previously obtained stable structure. All simulations start with the atomic density of the O_2_ molecules located over the surface at a distance of 4.5 to 5.5 Å from the uppermost Mg layer.

## Results and Discussion

### O_2_ Dissociative Adsorption on Mg(0001)

To reveal the formation of an MgO film, the surface reaction with O_2_ is investigated. Since the adsorption of O_2_ forms surface O atoms, the adsorption of one O atom on the *p*(4 × 4) Mg(0001) surface at 0.0625 monolayer coverage (ML) is studied first (1/16 ML). The adsorption energy (*E*_ad_) of one O atom is defined as *E*_ad_ = *E*_O/slab_ – 1/2*E*(O_2_) – *E*_slab_, where *E*_O/slab_ is the total energy of the slab with one adsorbed O atom, *E*_slab_ is the total energy of the bare slab, and *E*(O_2_) is the total energy of a free O_2_ molecule in the gas phase. To find the most stable site, all possible adsorption sites (Figure 1) on the surface, as well as in the first and second sublayers, were computed, and the corresponding adsorption energies and relative parameters are shown in Table 1.

**Table 1:** Adsorption Energy (*E*_ad_, eV), Shortest Mg–O Distance (*d*_O–Mg_, Å), and Bader Charge (δ_O_, e^–^) for Surface and Subsurface O Atom in Mg(0001) (0.0625 ML)

	surface	first sublayer			second sublayer		
Site	fcc	Oct1	TUH1	TUT1	Oct2	TUH2	TUT2
*E*_ad_	–3.94	–3.96	–4.45	–4.25	–3.82	–4.17	–4.06
d_O–Mg_	1.921	2.162	1.964	1.966	2.146	1.959	1.954
δ_O_	–1.68	–2.04	–1.78	–1.78	–1.97	–1.78	–1.78

It is found that O adsorption on the surface is only stable at the fcc site (−3.94 eV), while the O atom initially at the bridge and hcp sites is optimized into the first sublayer TUH1 site, which is most favorable (−4.45 eV, Figure 2a). The same result is found in the second sublayer, where the most stable site is TUH2 (−4.17 eV). Our results agree with previous DFT calculations.^28,30^ It is noted that the oxygen atoms located at all sites are negatively charged, indicating the strong electron transfer from magnesium atoms to oxygen atoms and the ionic character. The stronger adsorption energy at the TUH sites compared to the Oct sites can be attributed to the shorter Mg–O distance in the former than in the latter (Table 1). These show that at the initial stage, the O atom prefers to adsorb in the sublayer of Mg(0001) rather than on the surface.

**Figure 2:**
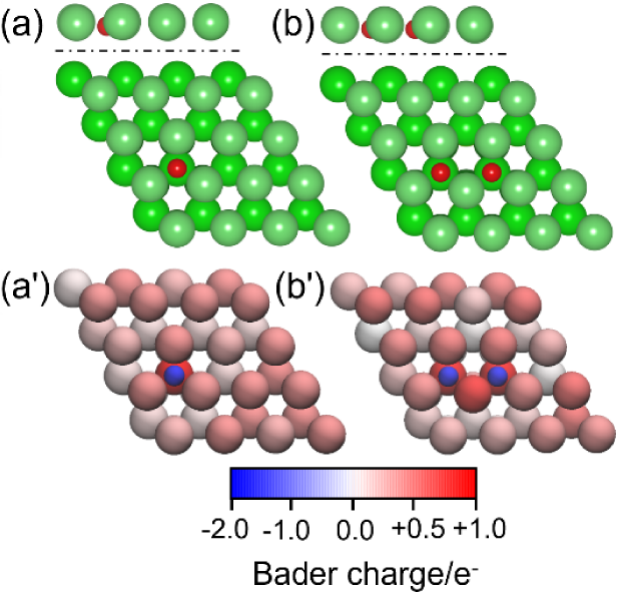
Most stable adsorption configurations (top: side view of the first layer, bottom: top view) of one O (a) and two O atoms (b) on the Mg(0001) surface; (a’) and (b’) are the corresponding Bader charge distributions.

Based on one O atom adsorption, we tested all possible sites for O_2_ adsorption (Table S2 and Figure S1) and found spontaneous dissociation. The most stable configuration (Figure 2b) has two O atoms at the neighboring TUH1 sites, and the adsorption energy (−9.08 eV) is more than twice that of one O atom adsorption (−8.90 eV) and that of two O atoms at the remote TUH1 sites (−8.89 eV, Figure S2a). This can be attributed to stronger O–Mg–O interaction due to the shorter Mg–O distance compared with that of one O atom adsorption (1.950 Å vs 1.964 Å). The adsorbed O atoms are reduced and negatively charged (−1.8 e), the Mg atoms between and just below the two O atoms are positively charged and have a charge of around 1, and other Mg atoms around are less positively charged (Figure 2b,b’). Such spontaneous dissociation resulting in neighboring configurations is also found in AIMD simulations (Figure 3). Starting with an O_2_ molecule at 5.5 Å over the surface, O_2_ arrives at the surface after less than 0.3 ps, and then dissociates spontaneously and penetrates the first subsurface, first with one oxygen atom at the TUH1 site and one oxygen atom at the hcp site, and finally with both oxygen atoms at the neighboring TUH1 sites after 0.4 ps. This configuration remains stable after 10 ps, in agreement with the DFT results.

**Figure 3:**
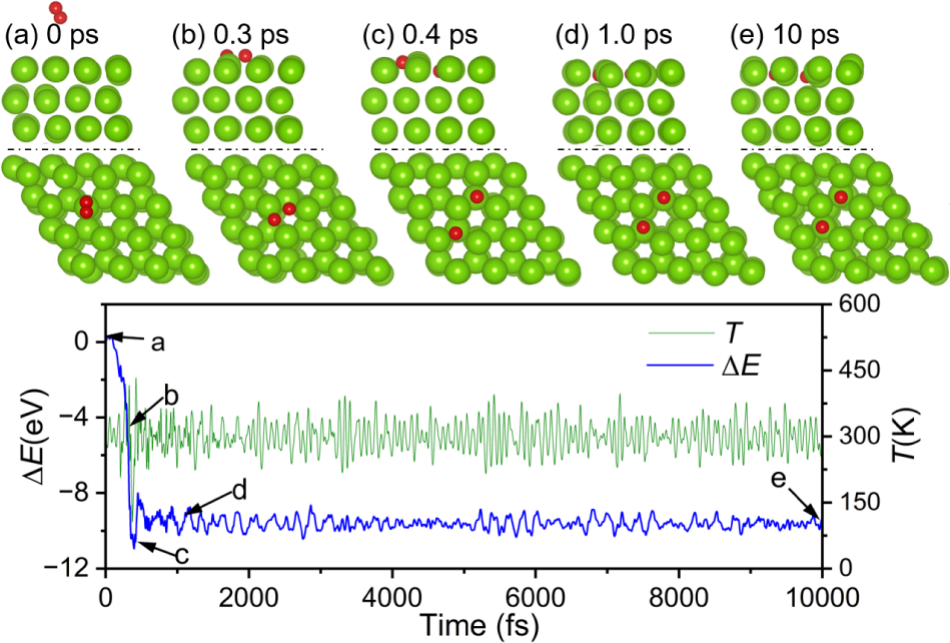
Trajectory of AIMD simulations (top: side view; bottom: top view) of the first O_2_ molecule reaction on Mg(0001) and the energy and temperature evolution.

### Diffusion of Adsorbed Oxygen

Since the diffusion of adsorbed oxygen atoms is another factor affecting the formation and thickening of the oxide film, we first computed the diffusion of one oxygen atom from the surface to the first subsurface, and then to the second subsurface (Figure 4). The possible paths are listed in Figure 4a.

**Figure 4:**
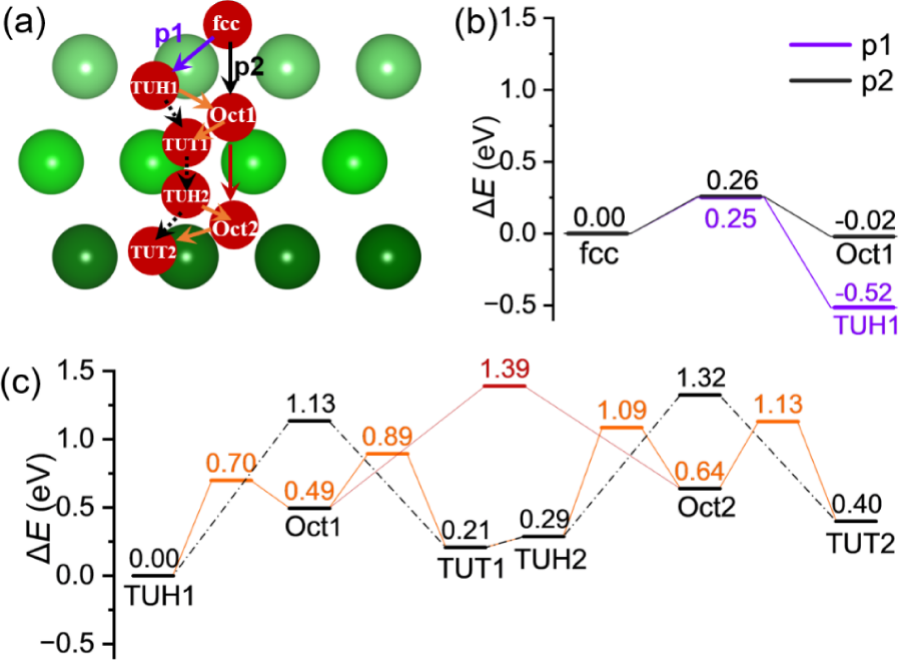
O diffusion pathways (a); potential energy surface of O diffusion from surface to first subsurface (b); and O diffusion from TUH1 to TUT2 site (c). (The colors in Figure 4bc are accordance to the color of the diffusion path in Figure 4a).

Since the surface-adsorbed O atom at the fcc site is less stable than that at the TUH1 site, it is expected that the diffusion should have a very low barrier. As shown in Figure 4b, the barrier from the surface fcc site to the subsurface TUH1 site (path 1) is very small (0.25 eV), and this step is highly exothermic by 0.52 eV, and the diffusion from the fcc site to the Oct1 site (path 2) has also close barrier (0.26 eV) but is much less exothermic (−0.02 eV). This is consistent with the spontaneous dissociation of one O_2_ adsorption and the subsequent penetration from the surface layer into the subsurface layer, as indicated by DFT calculations and AIMD simulations.

Next, the diffusion from the first subsurface to the second subsurface was calculated (Figure 4c). For the diffusion from the TUH1 site to the TUT1 site, the direct path has a higher barrier than the stepwise path (1.13 vs 0.70 eV). From the TUT1 site to the TUH2 site, the transition state was not located, and this step is only slightly endothermic, and it is merely the oscillation of an O atom between two neighboring tetrahedral sites.^43^ From the TUH2 site to the TUT2 site, the stepwise path has a lower barrier than the direct path (0.80 vs 1.03 eV). The transition from the Oct1 site to the Oct2 site has a barrier of 0.90 eV and is endothermic by 0.15 eV. Starting from the most stable TUH1 site in the first subsurface, the stepwise diffusion to the TUT2 site has an apparent barrier of 1.13 eV, indicating the kinetic difficulty of the diffusion.

### Oxide Film Formation

Based on these results, we computed the formation of a surface oxide film at increased oxygen exposure. The adsorption energy at high coverage has been computed based on the most stable adsorption configurations for one and two O atoms via stepwise addition of oxygen atoms (*n* = 1–16), and the stepwise adsorption energy (Δ*E*_ad_) is used to define the change in the adsorption energy upon additional adsorption. The stepwise adsorption energy is defined as Δ*E*_ad_ = *E*(O_n+1_/slab) - *E*(O_n_/slab) – 1/2 *E*(O_2_), where *E*(O_n_/slab) and *E*(O_n+1_/slab) are the total energies of the slab with *n*×O atoms and (*n*+1)×O atoms adsorption, respectively. The adsorption sites and stepwise adsorption energies are listed in Table S3.

Since the adsorption of 2 × O prefers neighboring oxygen atoms, we computed both aggregated and remote configurations and found that the adsorbed oxygen atoms prefer the aggregated configuration with the increase in coverage (Figure 5). For example, the first three adsorbed oxygen atoms (3 × O) prefer the neighboring sublayer TUH1 sites, the same as found by Francis et al.^30^ For 4 × O adsorption, the adsorption configuration of 3 × TUH1 and 1 × TUH2 is more stable than at 4 × TUH1 (Figure S2b) by 0.13 eV. For 5 × O adsorption, the adsorption configuration of 3 × TUH1 and 2 × TUH2 is more stable than that of 5 × TUH1 (Figure S2c) by 0.33 eV. For 6 × O adsorption, the adsorption configuration of 4 × TUH1 and 2 × TUH2 is more stable than that of 6 × TUH1 (Figure S2d) by 0.30 eV. Based on the 6 × O adsorption, the configuration of 5 × TUH1 and 2 × TUH2 for 7 × O adsorption is 0.36 eV more stable than the proposed hexagonal 7 × O island (6 × TUH1 sites and 1 × hcp site, Figure S2e) by Francis et al.^30^ For 8 × O adsorption, the configurations of 5 × TUH1 and 3 × TUH2 are more stable than those of 6 × TUH1 and 2 × TUH2 by 0.15 eV (Figure S2f). Therefore, one nucleation trend can be identified, i.e., preferring the TUH1 site and then the TUH2 site.

**Figure 5:**
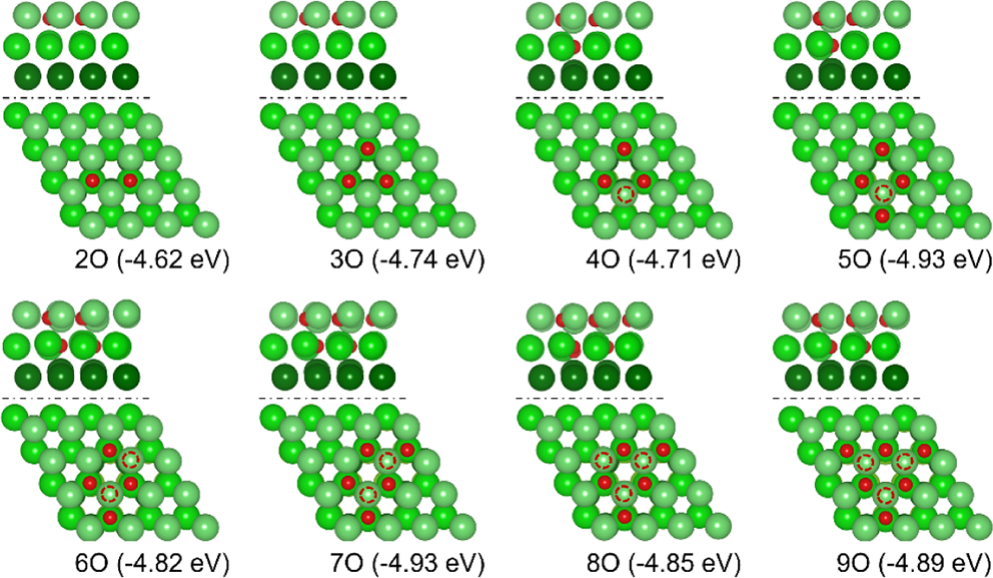
Side (top) and top (bottom; red circles indicate the presence of TUH2 sites) views of the most stable configurations and stepwise adsorption energy of n × O atoms (*n* = 2–9) on Mg(0001) (Mg/green, O/red).

Based on the most stable configuration of 2 × O adsorption from DFT and AIMD simulations, we performed AIMD simulations by putting another O_2_ molecule 5.5 Å directly over the oxygen-adsorbed surface sites (Figure 6a) and observed spontaneous dissociation. The O_2_ reaches the surface and dissociates after approximately 0.7 ps (Figure 6b). Subsequently, one O atom transfers to the hcp site just above the already existed TUH1 O atom, and another O atom locates on a neighboring TUH1 site after approximately 1 ps (Figure 6c). Then, the upper hcp O atom pushes the former TUH1 O atom into the Oct1 site quickly, and the hcp O atom moves down to the TUH1 site after approximately 1.2 ps (Figure 6d). The configuration with three O atoms located at the neighboring TUH1 sites and one O atom located at the Oct1 site remains stable after 10 ps (Figure 6e).

**Figure 6:**
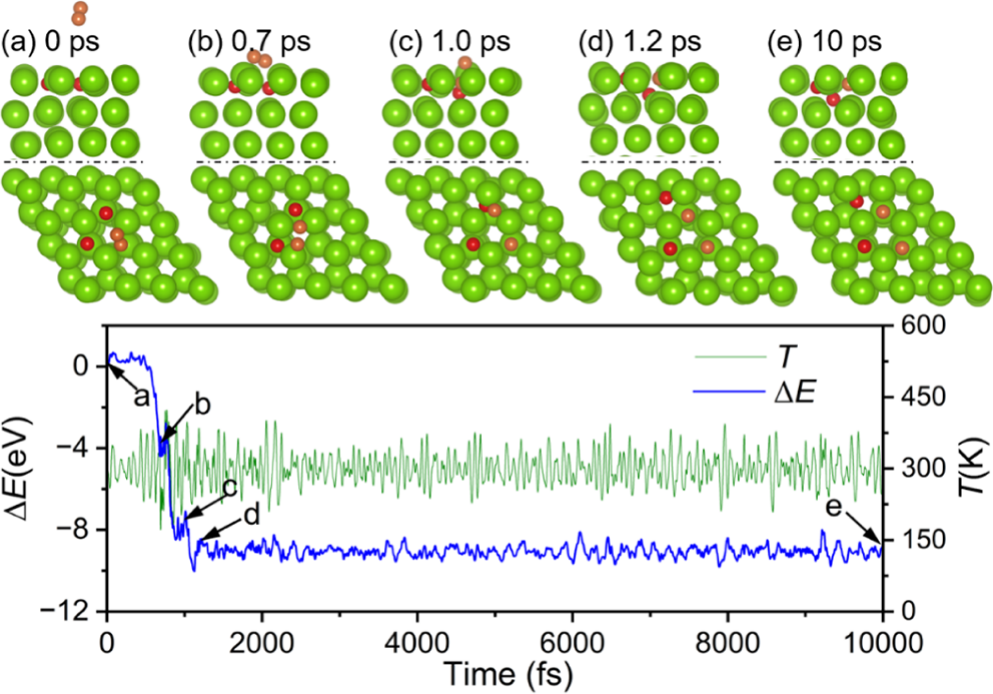
Trajectory of AIMD simulations (top: side view; bottom: top view) of the second O_2_ molecule reaction on 2xO adsorbed Mg(0001) and the corresponding energy and temperature evolution (Mg/green, O/red, the new coming O atoms are orange).

Similar results from AIMD simulations are found for additional O_2_ adsorption based on the 4 × O, 6 × O, 8 × O 10 × O, 12 × O, and 14 × O adsorption configurations (Figures S3–S7 and 7), i.e., the O_2_ molecule in the gas phase arrives at the surface very quickly and then dissociates spontaneously into surface oxygen atoms, which arrange into a stable adsorption configuration. It is also noted that with the increase in adsorbed oxygen atoms, the time of O_2_ from the gas phase to reach the surface becomes longer, such as for one additional O_2_ adsorption on 4 × O to 10 × O configurations. With the further increase in adsorbed oxygen atoms, not only molecularly activated but also dissociated metastable states are formed, and this can especially be observed for the additional O_2_ adsorption on 12 × O and 14 × O configurations. For the 14 × O configuration based on the 12 × O configuration from AIMD simulations, for example, the molecularly activated O_2_ configuration is stable between 0.7 and 6.3 ps, and then collapses to the dissociated configuration, which remains stable until 15 ps (Figure 7). However, the stable adsorption configuration from AIMD simulations is less stable than that from DFT. For 4 × O and 8 × O adsorption, for example, the AIMD configuration is less stable than that of DFT by 0.71 and 1.50 eV, respectively, indicating that AIMD configurations do not reach their thermodynamic stability; this can be explained by the high barrier of O diffusion.

**Figure 7:**
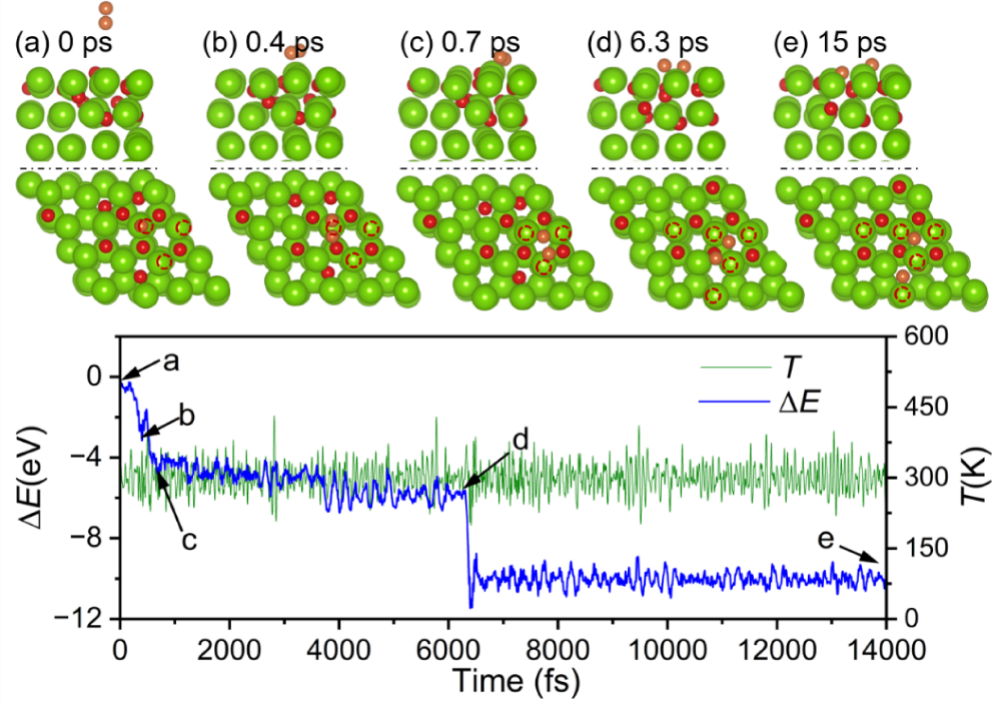
Trajectory of AIMD simulations (top: side view; bottom: top view) of the seventh O_2_ molecule reaction on 12 × O adsorbed Mg(0001) surface and right the corresponding energy and temperature evolution (Mg/green, O/red, the new coming O atoms are orange, red circle indicated there is an O atom just under the Mg atom).

Based on the DFT-computed trend of the TUH1 site preferring the TUH2 site, we computed the stepwise adsorption for 9 × O to 16 × O (Figure S8 and Table S3). From 2 × O to 16 × O, one can see that stepwise adsorption energies are close and do not show a decreasing trend, indicating the nonrepulsive interaction among these O atoms and the thermodynamic driving force for further oxygen adsorption. To check this possibility, we computed the full-coverage adsorption configurations for two (32 × O), three (48 × O), and six (96 × O) stoichiometric oxide layers and found a further increase in the average adsorption energy (−4.96, −5.10, and −5.17 eV, respectively), indicating the potential for full oxidation. All of these adsorption configurations show the termination of magnesium at the first layer, indicating the possibility for additional adsorption of oxygen atoms despite the surface stoichiometry.

Next, *ab initio* atomistic thermodynamics (Section S1) is applied to study the surface oxidation as a function of oxygen chemical potential (Figure 8).^44,45^ It shows that under practical conditions, the metallic magnesium surface is not stable and can be easily oxidized by molecular O_2_ at room temperature, even at negligible partial pressure (*p* = 10^–190^ atmosphere). Based on the heat of formation of the bulk oxide at *T* = 0 K (Δ*H*_f_ = −6.18 eV for MgO,^46^ the bulk oxide will always be a stable phase at Δμ_O_ higher than this limit. Thus, thermodynamically, full oxidation is favored.

**Figure 8:**
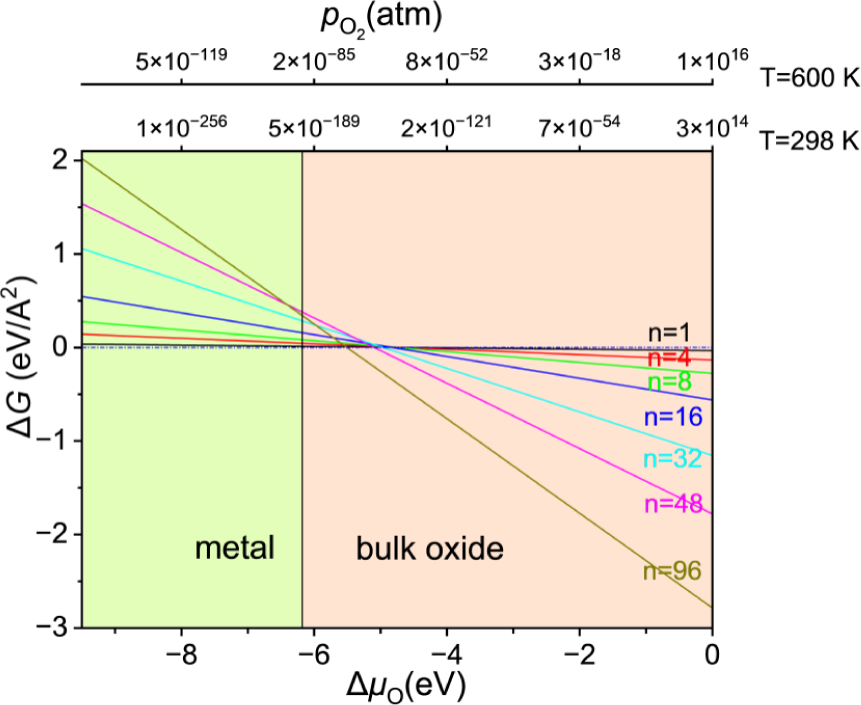
Computed Gibbs free energy of the O surface oxide on the Mg(0001) surface energy as a function of change of oxygen chemical potential (Δμ_O_) (The dependence of Δμ_O_ on pressure scale at *T* = 298 and 600 K is shown for clarity. In the bottom of the figure, the stable type in the corresponding range of O potential is listed and indicated by the shaded regions).

### Surface Peroxides

Since the oxidation is favored thermodynamically and the chemisorbed oxygen under the surface layers grows laterally and vertically, oxidized layers will be formed, and further oxidation will thicken the oxidized layers.^19^ We computed the adsorption and diffusion of O_2_ from the gas phase on the two-layer stoichiometrically oxidized surface with 32 oxygen atoms (Figure S8), and these results are shown in Figure 9 and Table S4.

**Figure 9:**
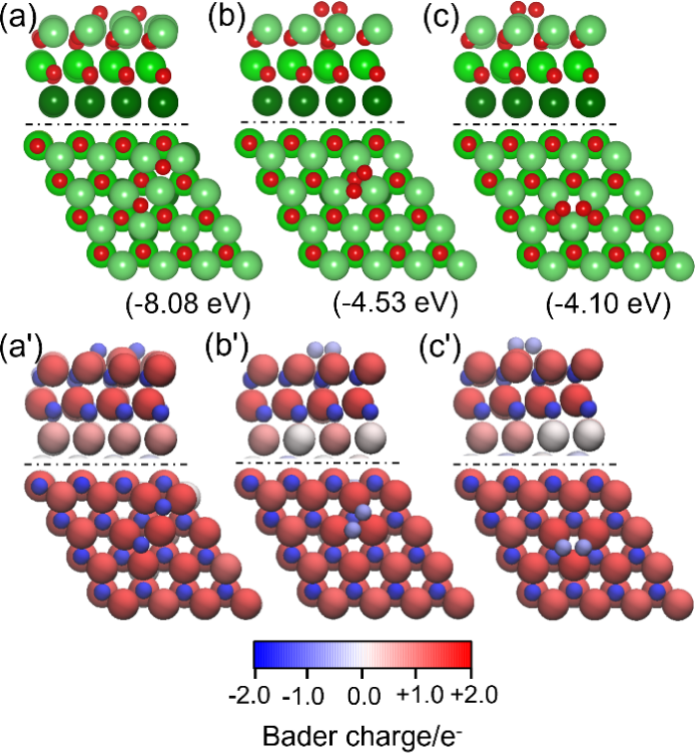
Adsorption configurations (top: side view, bottom: top view) of two O atoms adsorbed on the fcc sites (a), bridge sites (b), and bridge site 2 (Mg/green, O/red) on 32 × O atoms oxidized surface. (c) and corresponding Bader charge distributions (a’-c’).

It is found that the most stable configuration has two oxygen atoms at the fcc sites with an adsorption energy of −8.08 eV (Figure 9a), close to that of the case on the clean surface (−7.88 eV), and only slightly lower than that at the neighboring TUH1 sites (−9.08 eV) on the clean surface. This indicates the strong adsorption despite the stoichiometrically two-layer oxidized surface, and this can be attributed to the exposed magnesium termination. Therefore, further adsorption of more oxygen atoms on such an oxidized surface is thermodynamically possible.

In addition to the dissociative adsorption at the surface fcc sites, the molecularly adsorbed O_2_ with an elongated O–O distance (1.56/1.54 Å, Figure 9b,c, compared to 1.21 Å of free O_2_) has much lower adsorption energy at the bridge sites (−4.53 and −4.10 eV). It is noted that the dissociative oxygen atoms (−1.58 e for each O atom) are more negatively charged than molecularly adsorbed O_2_ (−1.70 e in total), which can be considered as a peroxide anion state (O_2_^2–^). Nevertheless, activated O_2_ can decompose easily with a negligible barrier of 0.1 eV at low oxygen coverage.

With the further increase in oxygen coverage (Figure 10), the dissociated configuration is more stable than the molecularly activated configuration for 2 × O_2_ adsorption (−17.66 vs −9.32 eV); however, this energy difference vanishes for 4 × O_2_ adsorption (−19.41 vs −18.63 eV). For the adsorption of 5O_2_ and 8O_2_, on the contrary, the molecularly activated configuration becomes more stable than the dissociated configuration (−20.84 vs −18.03 and −23.39 vs −10.63 eV, respectively) by 2.81 and 12.76 eV, respectively. This shows that there should be equilibrium between the dissociated and molecularly activated configurations for 4 × O_2_ adsorption. To verify this proposal, we computed the adsorption of 4 × O_2_ molecules on the surface (Figure 11b,c) and found that the mixed adsorption configuration with 4O atoms and two molecularly activated configurations (4O+2O_2_) is more stable than the fully dissociated configuration (−20.82 vs −19.41 eV) by 1.41 eV, and the mixed adsorption configuration with 6O atoms and one molecularly activated configuration (6O+O_2_, −20.18 eV) by 0.64 eV.

**Figure 10:**
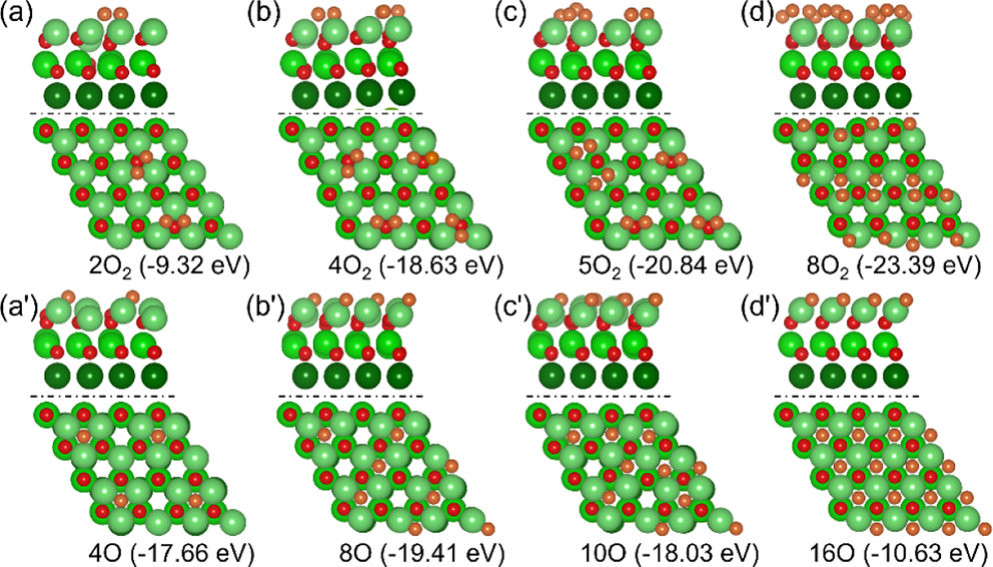
Adsorption configuration (Mg/green, O/red), adsorption energy (*E*_ad_) for 2 × O_2_ molecules (a, a’); 4 × O_2_ molecules (b, b’); 5 × O_2_ molecules (c, c’); 8× O_2_ molecules (d, d’) on 32xO atoms oxidized surface; (a-d) are molecularly activated configuration and (a’-d’) are dissociated configuration.

**Figure 11:**
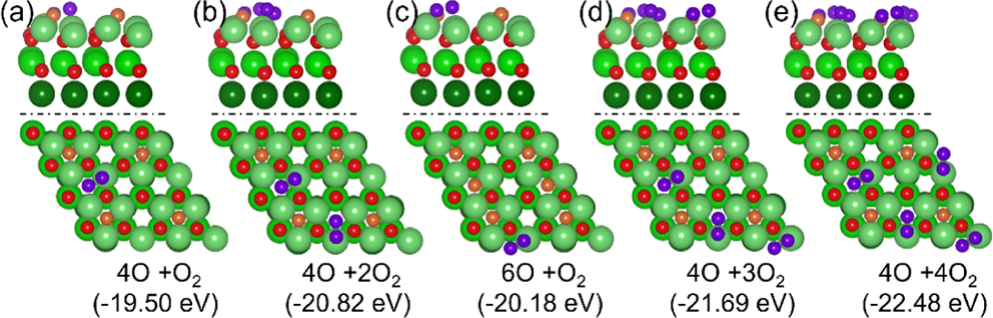
Adsorption configuration, adsorption energy (*E*_ad_) for 4O+O_2_ (a); 4O+2O_2_ (b); 6O+O_2_ (c); 4O+3O_2_ (d); 4O+4O_2_ (e) on 32O atoms oxidized surface (Mg/green, O/red, O_2_ on the surface is marked as purple).

Based on the adsorption configuration of 4O, we computed the adsorption configuration of 4O+xO_2_ and found that the stepwise adsorption energy of 4O+O_2_, 4O+2O_2_, 4O+3O_2_ and 4O+4O_2_ (as shown in Figure 11) is −1.83, −1.33, −0.87, and −0.78 eV, respectively. The corresponding stepwise desorption temperatures at 1 × 10^–6^ Torr are 607, 453, 311, and 285 K, respectively. At 1 atm, the desorption temperatures are 836, 623, 428, and 392 K, respectively, indicating the stable mixed adsorption configuration of 4O+4O_2_.

For the molecularly activated configurations, the O–O bond lengths are around 1.5 Å, much longer than that of free O_2_ (1.21 Å), while close to that of magnesium peroxide (MgO_2_), 1.51 Å^47^ and the computed O–O stretching frequency is between 704 cm^–1^ (1.56 Å) and 1112 cm^–1^ (1.34 Å).

Therefore, it should be possible to detect magnesium peroxide on the surface with increasing O_2_ exposure. The magnesium peroxide might be responsible for the XPS signal at ∼533 eV.^24^ Since the computed desorption temperature of the peroxide state is very high, the peroxides upon annealing at 700 K dissociate and the resulting oxygen atoms diffuse into the sub- and deep layers, and thickening of the oxide layer will occur accordingly.

To further investigate the oxidation film thickening after two-layer stoichiometric full oxidation (32 × O), AIMD simulations of further O_2_ adsorption and dissociation on the top two-layer fully oxidized Mg(0001) surface at 300 K were investigated. The simulations began with four O_2_ molecules (4 × O_2_) about 5.5 Å randomly over the surface (Figure 12a). Around 0.4 ps, one O_2_ molecule reaches the surface and starts to dissociate (Figure 12b) and becomes completely dissociated after approximately 0.6 ps, with both oxygen atoms are located on the two hcp sites (Figure 12c). At the same time, two other O_2_ molecules also reached the surface. Subsequently, the dissociated oxygen atoms pushed the oxygen atoms in the oxidized layer at the TUH1 sites just below (marked in purple) to the Oct1 sites (Figure 12d). Then, the last O_2_ molecule also reached the surface (Figure 12e). With the further increase in time (Figure 12f–h), the oxygen atoms at the Oct1 sites fluctuated downward, and the magnesium atoms nearby moved upward, resulting in disorder among the atoms in the oxidized layer. The distorted AIMD structure was further refined using DFT (Figure S9) and no significant changes were observed. The coordination number of three O atoms (marked with numbers in Figure S9) near the upcoming Mg atoms was five, and the Mg–O bond lengths of these three O atoms ranged from 1.97 to 2.25 Å, longer than the Mg–O bond lengths (around 1.96 Å) of the 4 coordinated O atoms. The oxidation layer became distorted, with the oxygen atoms shifting downward and the magnesium atoms shifting upward. With the increase of dissociated oxygen atoms and the diffusion of oxygen atoms into the surface, the structure becomes amorphous, as in the oxidation of crystalline Si to SiO_2_.^48^ This might explain the observed disappearance of the LEED pattern during the further oxidation process.^20^ Finally, one O_2_ molecule dissociated and became surface oxygen atoms, while the three other O_2_ molecules remained on the surface and formed molecularly activated configurations with O–O distances within 1.50–1.60 Å. Once again, AIMD simulations did not converge to a stable thermodynamic adsorption configuration.

**Figure 12:**
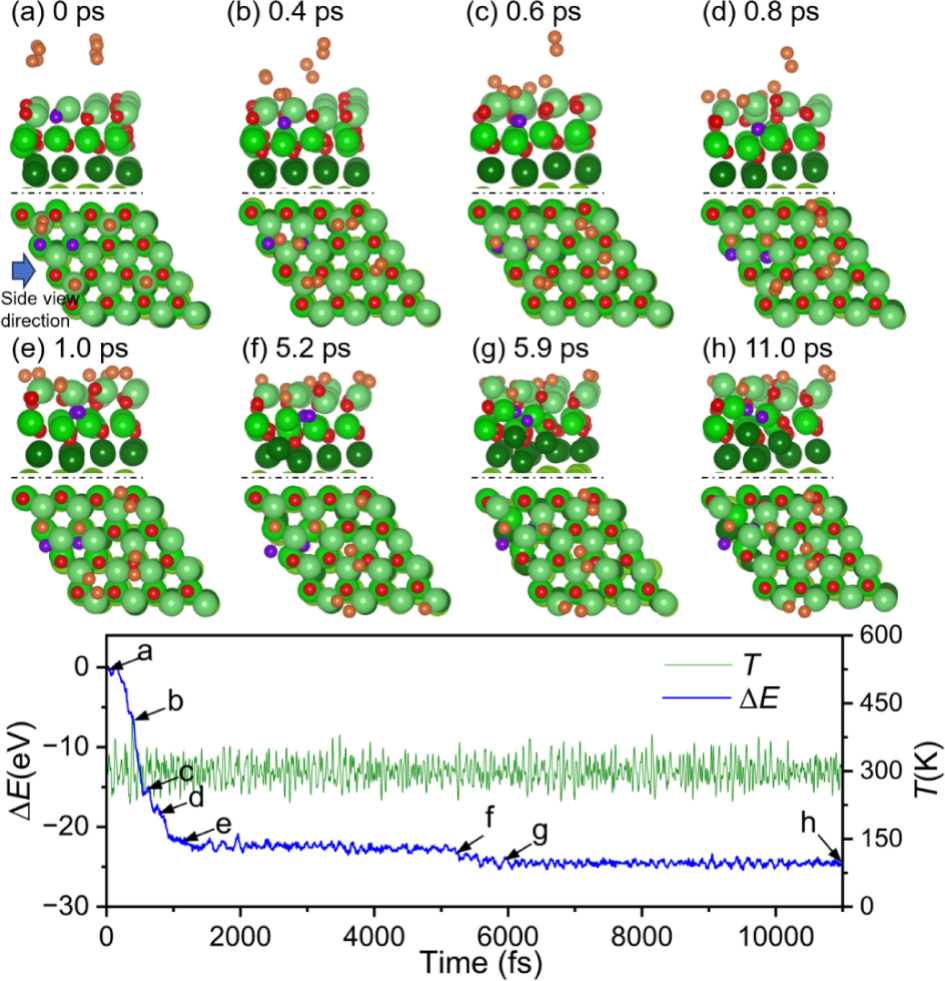
Trajectory of AIMD simulations (top: side view; bottom: top view) of the four O_2_ molecule reaction on 32 × O adsorbed Mg(0001) surface and bottom the corresponding energy and temperature evolution (Mg/green, O/red, the new coming O atoms are orange).

## Discussion

Based on these results, the oxidation process can be described in Figure 13. At the initial stage, oxygen molecules dissociate spontaneously and penetrate the subsurface, accompanied by electron transfer from magnesium atoms to oxygen atoms. With the further increase in oxygen exposure, the newly dissociated oxygen atoms can push oxygen atoms at the TUH1 sites to the neighboring TUH2 sites easily, although the diffusion of oxygen atoms from TUH1 sites to deeper sites has a high barrier. The resulting electrostatic attractive O–Mg–O interaction facilitates the formation of oxidized islands, which grow both laterally and vertically, and finally, the first two layers are oxidized. During this process, the oxygen atoms are located in the subsurface, and the oxide islands just commensurate with the host lattice, while the Mg(0001) surface structure remains almost undistorted, in accordance with the almost unchanged LEED pattern of the surface during the initial oxidation process.^28^

**Figure 13:**
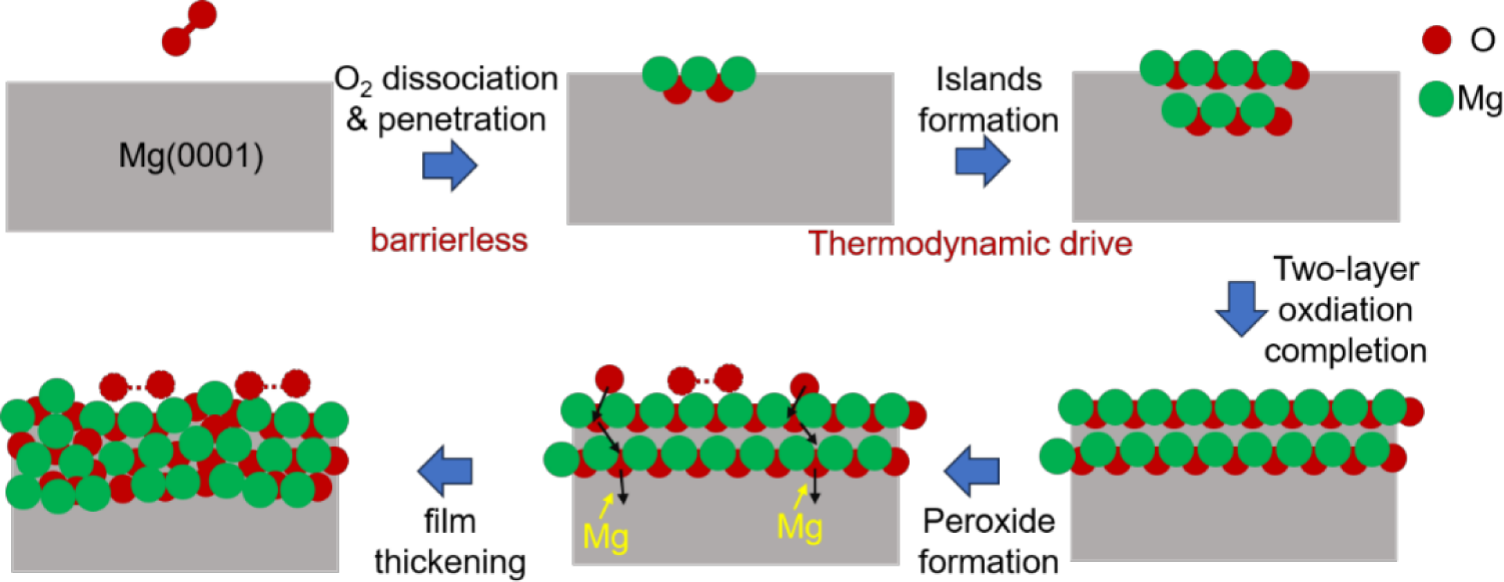
Oxidation process according to the DFT calculation.

When the first two layers of oxidation have been completed, the Mg atoms in these two oxidized layers are positively charged, and the O atoms are negatively charged. The attractive interaction between the negatively and positively charged electronic structures makes this structure stiffer, and the electron donation ability of Mg is limited. Then, the subsequent O_2_ molecule approaches this surface and receives electrons from the surface more slowly. The rate of O_2_ dissociation is slowed down, and both dissociative adsorption (O) and molecular adsorption (O_2_) occurred on the surface. The molecular adsorption state (activation O_2_ state, O_2_^2–^) on the surface leads to the occurrence of the XPS signal at high binding energy.^24^ The further uptake of both oxygen atoms and molecular O_2_ forms stable mixed adsorption configurations (4O+xO_2_, x = 1–4) on the surface. The O atoms diffuse deeper, and at the same time, the unoxidized Mg in the down layer will be attracted to the bottom of the oxidation layer, and thus leads the surface distortion heavily, and there will be a gap between the unoxidized layer and the oxidized layer. The surface distortion will lead to the diminishment of the LEED signal, which is in accordance with the experimental results .^20^ In addition, not only the dissociation of O_2_ molecules but also the diffusion of O atoms slows down during this phase, both lead to a decrease in the oxidation rate, which is similar to the oxidation of Si to SiO.^48^ However, unlike the direct diffusion of O or O_2_ during the oxidation of Si, in this case, the dissociated O atoms occupy surface sites and drive the O atoms at these sites downward.

The oxidized surface will eventually convert into the more stable rock salt structure from the point of energy; this will lead to the reoccurrence of the LEED signal, although the phase transition is beyond the range accessible to our simulation.

Due to time and length scale limitations in our AIMD simulation, it is difficult to cover the long oxidation evolution (e.g., phase transfer) observed in the experiment. In this study, however, we have captured microkinetic processes such as O_2_ adsorption/dissociation (including peroxide formation) and O atom diffusion at the initial stage, as well as the film thickening process, using AIMD, in good agreement with the experimental results. The computed thermodynamic data support the conclusion that the oxidized surface will eventually be converted into the more stable rock salt structure. Furthermore, the actual oxidation of magnesium is a complex process, and actual defects (e.g., steps, vacancies) on the magnesium surface, as well as moisture and other substances in the air, can affect the oxidation process, which will be investigated in our future work.

## Conclusion

To provide insight into the oxidation process of metallic magnesium surfaces for corrosion protection, the adsorption and dissociation of molecular O_2_ on the Mg(0001) surface have been investigated based on systematic DFT computations and AIMD simulations upon the increase of O_2_ exposure. Our atom-level study provides insight into the oxidation process, including oxygen incorporation, monolayer completion, surface peroxide formation, and film thickening as molecular O_2_ exposure.

At the initial stage of O_2_ exposure, molecular O_2_ prefers spontaneous dissociative adsorption on the surface, and the dissociated oxygen atoms penetrate the subsurface, preferably at the neighboring subsurface tetrahedral sites, resulting in the formation of oxide islands due to electrostatic attractive O–Mg–O interactions at high O_2_ exposure. The strong stepwise dissociative adsorption energy reveals the thermodynamic driving force for full oxidation, as also proven by *Ab initio* atomistic thermodynamics analysis, although the diffusion of O atoms in the subsurface suppresses full oxidation.

With the increase in the level of exposure to O_2_, the islands grow rapidly and epitaxially, resulting in the formation of two stoichiometrically oxidized layers, in line with the experimental proposal. During this process, the oxygen atoms become commensurate with the host lattice, and the Mg(0001) surface structure remains almost undistorted, in agreement with the nearly unchanged LEED pattern of the surface during the initial oxidation process. It is interesting to note that the surface of the two stoichiometrically oxidized layers can further uptake O_2_ adsorption, resulting in dissociative adsorption (O), molecular adsorption (O_2_) and a mixed stable adsorption configuration of 4O+xO_2_ (x = 1–4). Such molecularly adsorbed O_2_ configurations have been identified as the peroxide state (O_2_^2–^), and this may be related to the satellite signal peak at higher binding energy, which appears after the main peak with the increase of O_2_ exposure, as observed in XPS measurements.

These stable peroxide states are stable and prefer dissociation over desorption at high temperatures, and further O_2_ dissociation and diffusion on the surface result in the thickening of the oxide layers. This explains the gradual disappearance of the higher binding XPS signal with the increase in temperature. Furthermore, further thickening of the oxide layers causes surface deformation, which agrees well with the LEED signal disappearance.

## Supplementary Material



## Notes

Ab initio thermodynamics method; adsorption configuration; snapshots of AIMD simulations and the corresponding energy and temperature evolution during simulation; adsorption sites, adsorption energy, average adsorption energy, and stepwise adsorption energy under different coverages­(PDF)
