# Supplementary material for: O2 Dissociative Adsorption on Mg(0001)—Surface Oxidation, Peroxide Formation, and Oxide Layer Thickening
Source: ACS Omega. 2025 Jun 2;10(23):24907–15. doi: 10.1021/acsomega.5c02179 (PMC12177582; doi:10.1021/acsomega.5c02179)
Supplement: Supplementary file 1 [file ao5c02179_si_001.pdf]

Supporting Information

O_2 dissociative adsorption on Mg(0001) - surface oxidation, peroxide formation and oxide layer thickening

Yunyan Han,[a, b] *and Haijun Jiao*[b]*

a. Key Laboratory of Eco-functional Polymer Materials of the Ministry of Education, College of Chemistry and Chemical Engineering, Northwest Normal University, Lanzhou, 730070, China.

b. Leibniz-Institut für Katalyse e.V. (LIKAT), Albert-Einstein-Str. 29A, 18059, Rostock, Germany.

---

*Corresponding author.
E-mail: Haijun.Jiao@catalysis.de

Table of Contents

Table of Contents S2
1. *Ab initio* thermodynamics method S2
2. O_2 Desorption temperature calculation S5
3. Figure S1-S8. .... S6
4. Tables S1-S5 .... S15
References S19

1. *Ab initio* thermodynamics method

The surface free energy (γ) of a surface can be described as in Eq. 1, in which G is the Gibbs free energy of a solid surface, A is the surface area of the slab surface, $\mu_i(T, p)$ is the chemical potential of the species i, n_i is the number of the ith type species.

$$\gamma(T,p) = \frac{1}{2A}\left[G - \sum_i n_i\mu_i(T,p)\right] \qquad \text{Eq.1}$$

The surface free energy of the clean Mg(0001) surface can be written as in Eq.2.

$$\gamma_{clean}(T,p) = \frac{1}{2A}\left[G_{Mg}^{clean}(T,p) - n_{Mg}\mu_{Mg}(T,p)\right] \qquad \text{Eq.2}$$

To the periodic slab, only one surface is used to calculate the oxygen adsorption. Therefore, the surface energy of the fixed surface should be subtracted when calculating the surface energy of the O adsorption surface. The surface energy of the Mg(0001) surface with n O atoms adsorption can be written as in Eq.3.

$$\gamma_{Mg}(T,p,nO) = \frac{1}{A}\left[G_{Mg}(T,p,nO) - n_{Mg}\mu_{Mg}(T,p) - n_O\mu_O(T,p)\right] - \gamma_{clean}(T,p) \qquad \text{Eq.3}$$

Thus, the change of surface free energy after O adsorption can be described as in Eq.4.

$$\Delta G = \gamma_{Mg}(T,p,nO) - \gamma_{clean}(T,p) = \frac{1}{A}\left[G_{Mg}(T,p,nO) - G_{Mg}^{clean}(T,p) - n_O\mu_O(T,p)\right] \qquad \text{Eq.4}$$

$$\mu_O(T,p) = \frac{1}{2}E_{O_2}^{total} + \Delta\mu_O(T,p) \qquad \text{Eq.5}$$

Combing Eq.4 and Eq.5, we can obtain,

$$\Delta G \approx \frac{1}{A}\left[E_{Mg,nO}^{total} - E_{Mg,clean}^{total} - \frac{n_O}{2}E_{O_2}^{total} - n_O\Delta\mu_O(T,p)\right] \qquad \text{Eq.6}$$

The correlation between $\Delta\mu_O(T,p)$ and p_{O_2} can be expressed in the following equation:

$$\Delta\mu_O(T,p) = \Delta\mu_O(T,p^\theta) + \frac{1}{2}k_BT\ln\left(\frac{p}{p^\theta}\right) \qquad \text{Eq.7}$$

$\Delta\mu_O(T,p^\theta)$ can be determined using tabulated enthalpy and entropy values at standard pressure $p^\theta = 1\ atm$.

$E_{Mg,nO}^{total}$ is the total energy of the Mg(0001) surface adsorbed with n × O atoms with ZPE correction, $E_{Mg,clean}^{total}$ is the total energy of the clean Mg(0001) surface, and $E_{O_2}^{total}$ is the total energy of O_2 gas with ZPE correction. All these three parameters are calculated using VASP. Thus, we can directly plot the

ΔG for each model (Mg(0001) surface with different number of O atoms) as a function of oxygen chemical potential ($\Delta\mu_O(T,p)$) according to Eq.6.

Under given temperature (T), the O_2 pressure (p) can be calculated under different oxygen chemical potential ($\Delta\mu_O(T,p)$) according to Eq.5. (The O_2 pressure under 298 K and 600 K corresponding to the $\Delta\mu_O(T,p)$ are indicated in the top of Fig.8.)

When the bulk oxide becomes thermodynamically more favorable than an equivalent amount of bulk metal and gas phase oxygen: $G_{\mathrm{MgO}} \leq G_{Mg} + \mu_O$, where G_{MgO} is the Gibbs free energy per formula unit of the oxide bulk. Using Eq.5, we get $\Delta\mu_O \geq G_{\mathrm{MgO}} - G_{Mg} - \frac{1}{2}E_{O_2}^{total} = \Delta H_f(T = 0\ K)$, where ΔH_{f} (T=0 K) is the heat of formation of the bulk oxide at $T = 0$ K. For any $\Delta\mu_{\mathrm{O}}$ higher than this limit, the bulk oxide will always be the stable phase.

2. O_2 Desorption temperature calculation

When n×O_2 molecules adsorbed on 32×O atoms oxidized magnesium surface (Mg_32O) forming $Mg\_32O.2nO$, the formular can be writen as follow: $Mg\_32O + n(O_2)(g) = Mg\_32O.2nO$

The Change in Gibbs free energy can be descirbed in Eq.8,

$$\Delta G_{ad} = G_{Mg\_32O.2nO}(T,p,nO) - G_{Mg\_32O}(T,p) - G_{nO_2}$$

$$\approx E^{total}_{Mg\_32O.2nO} - E^{total}_{Mg\_32O} - n\mu_{O_2}(T,p) \qquad \text{Eq.8}$$

$E^{total}_{Mg\_32O.2nO}$ is the total energy of the 32×O atoms oxidized magnesium surface adsorbed with n × O atoms with ZPE correction, $E^{total}_{Mg\_32O}$ is the total energy of the 32×O atoms oxidized magnesium surface, All these three parameters are calculated using VASP.

$$\mu_{O_2}(T,p) = E^{total}_{O_2} + \Delta\mu_{O_2}(T,p^0) + \frac{1}{2}k_B T ln(p/p^0) \quad \text{Eq.9}$$

$E^{total}_{O_2}$ is the total energy of O_2 gas with ZPE correction. $\Delta\mu_{O_2}(T,p^0)$ can be determined using tabulated enthalpy and entropy values at standard pressure $p^{\theta} = 1\ atm$.

Thus, combing Eq.8 and Eq.9, the desorption temperature (T) under differnt pressure (p) can be get according to the equation when $\Delta G_{ad} = 0$.

3. Figure S1-S9

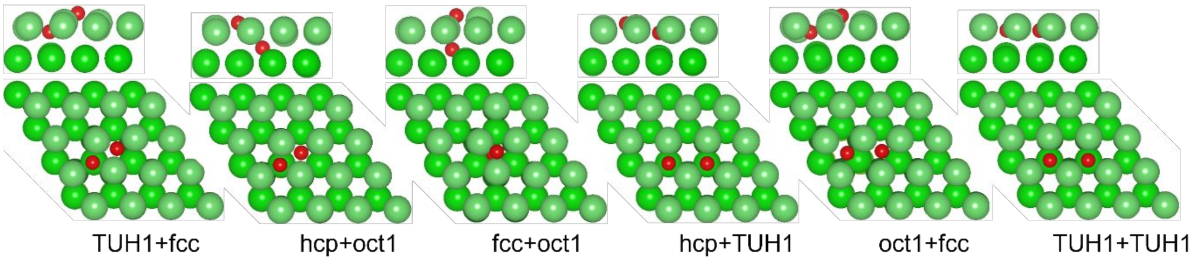

Fig. S1. Adsorption configurations after O_2 decomposition.

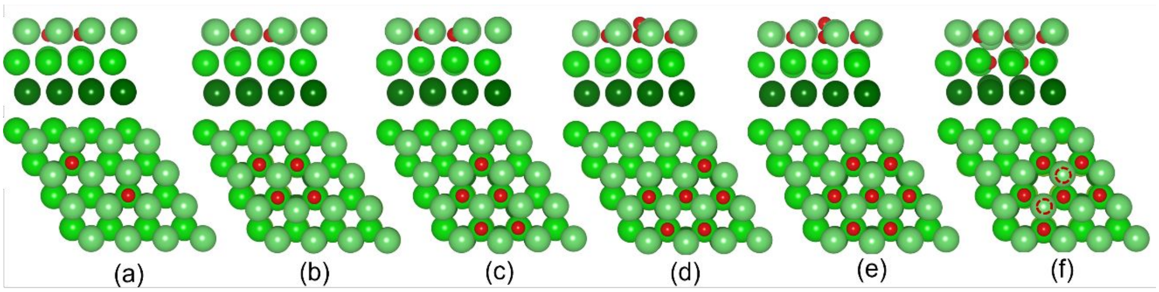

Fig. S2. Adsorption configurations for n×O atoms adsorption: (a) two O atoms at the remote TUH1 sites; (b)four O atoms at TUH1 sites; (c) five O atoms at TUH1 sites; (d) six O atoms at TUH1 sites; (e) six O atoms at TUH1 sites and one O atom at fcc site; (f) six O atoms at TUH1 sites and two O atoms at TUH2 sites.

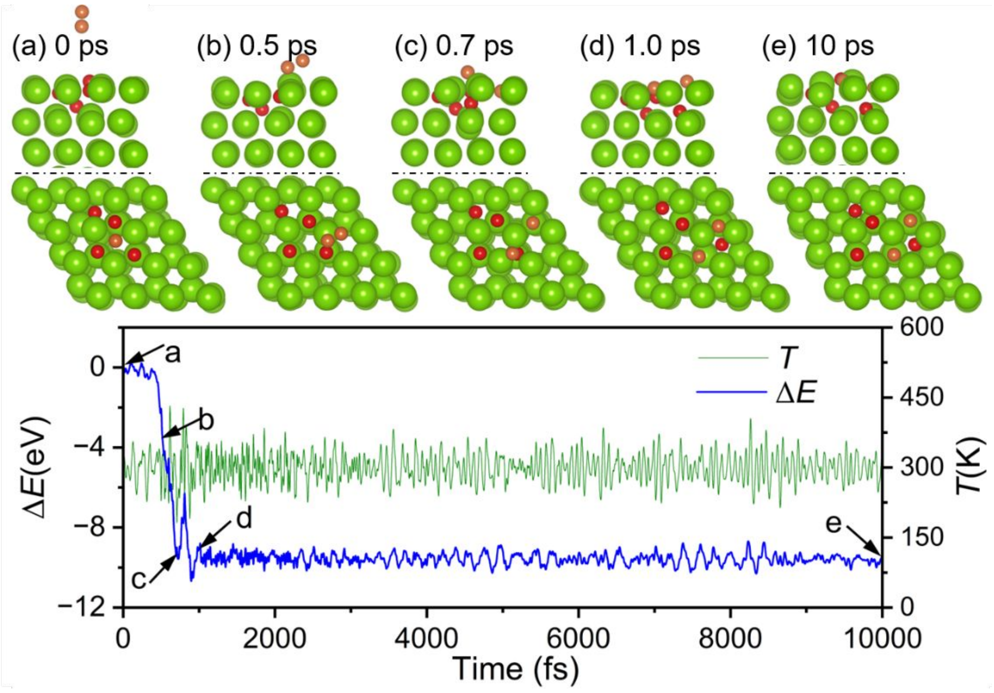

Fig. S3. Snapshots of AIMD simulations (top: side view; bottom: top view) of the third O_2 molecule reaction on Mg(0001) surface and the corresponding energy and temperature evolution during simulation.

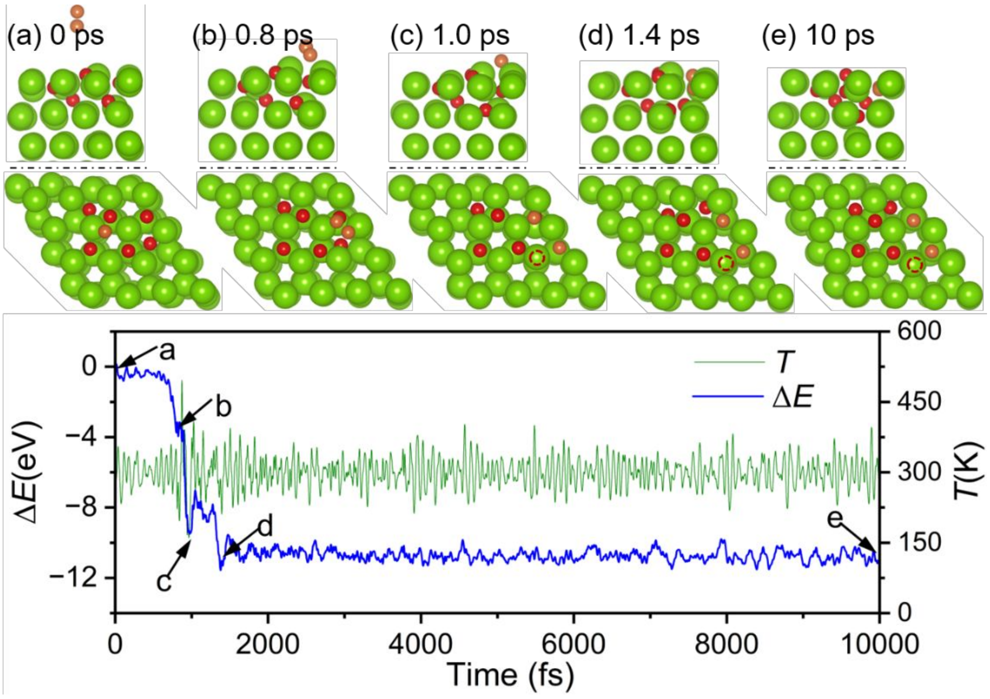

Fig. S4. Snapshots of AIMD simulations (top: side view; bottom: top view) of the fourth O_2 molecule reaction on Mg(0001) surface and the corresponding energy and temperature evolution during simulation.

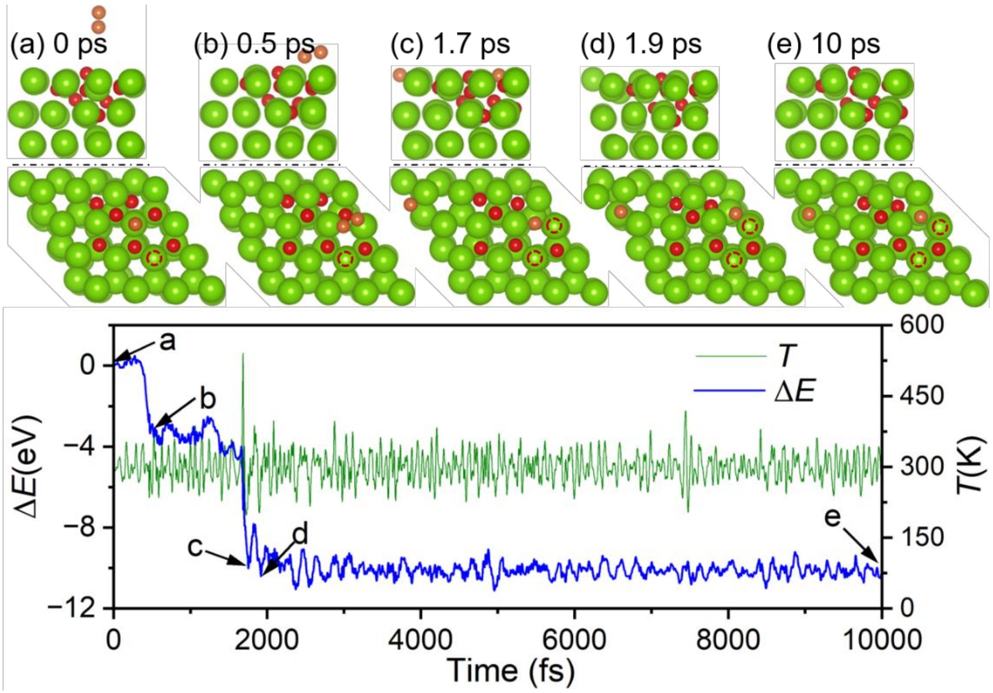

Fig. S5. Snapshots of AIMD simulations (top: side view; bottom: top view) of the fifth O_2 molecule reaction on Mg(0001) surface and the corresponding energy and temperature evolution during simulation.

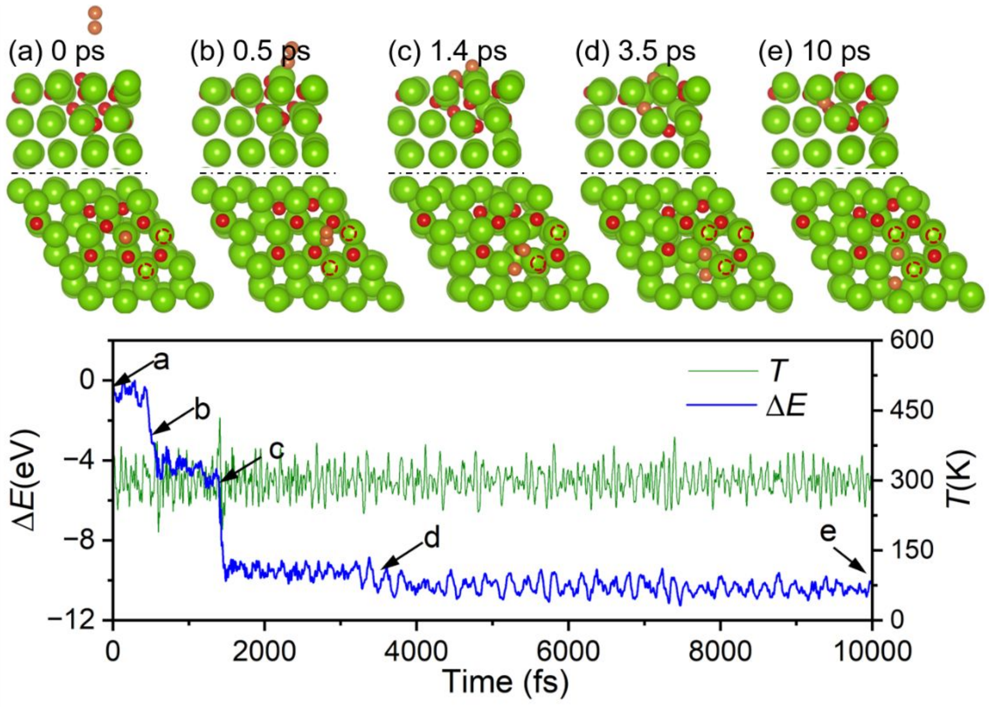

Fig. S6. Snapshots of AIMD simulations (top: side view; bottom: top view) of the sixth O_2 molecule reaction on Mg(0001) surface and the corresponding energy and temperature evolution during simulation.

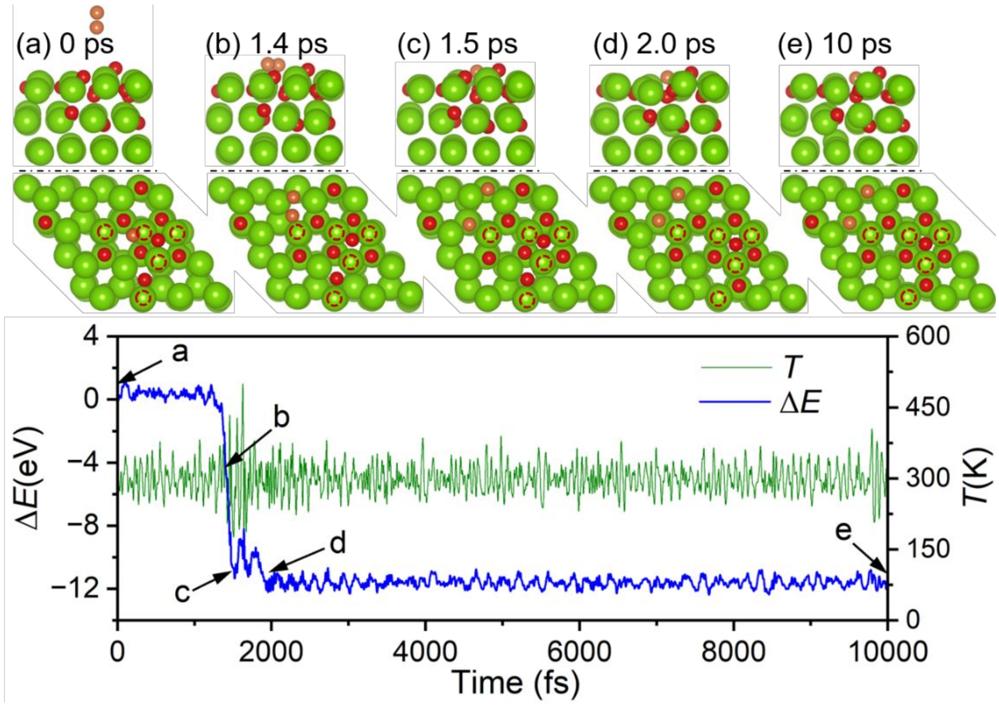

Fig. S7. Snapshots of AIMD simulations (top: side view; bottom: top view) of the eighth O_2 molecule reaction on Mg(0001) surface and the corresponding energy and temperature evolution during simulation.

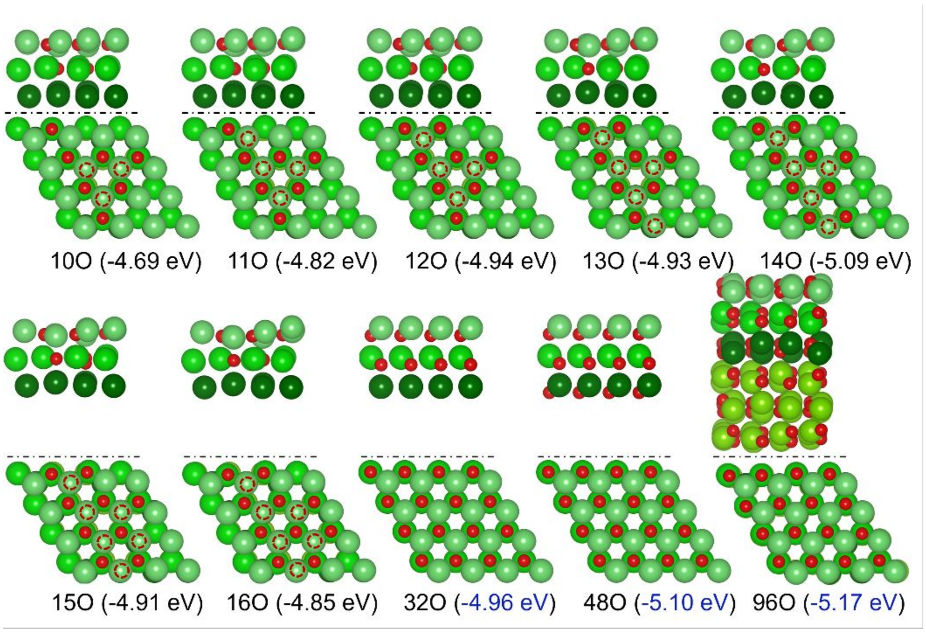

Fig. S8. Side (top) and top (bottom; red circles indicate O at the TUH2 sites) views of the most stable configurations and stepwise adsorption energy of n×O atoms (n = 10-16), as well as configurations and average adsorption energy of 32×O atoms (top two-layer), 48×O atoms (top three-layer) and 96×O atoms (fully oxidized) on Mg(0001) (Mg/green, O/red).

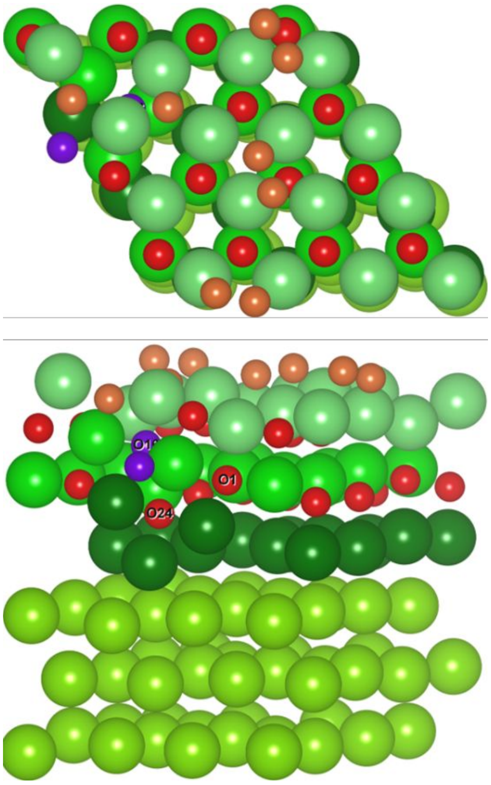

Fig. S9. Side (bottom) and top (top) view of the distorted structure after AIMD simulation of four O_2 molecule reaction on 32×O adsorbed Mg(0001) surface after DFT relaxation.

4. Tables S1-S5

Table S1. Relaxation of outermost layers at hcp Mg(0001).

	Δ_{12}(%)	Δ_{23}(%)	Δ_{34}(%)
This work	0.92	-0.05	-0.49
Experiment[1] (130 K)	1.96±0.5	0.2±0.6	0.0±0.9

Table S2. O_2 adsorption configuration before and after optimization, adsorption energy (E_{ad}, eV), O-O distances ($d_{O\text{-}O}$, Å) and O-Mg distance ($d_{O\text{-}Mg}$, Å) of the final adsorption state for O dissociation on Mg(0001) surface.

Initial site		Final site	E_{ad}	$d_{O\text{-}O}$	$d_{O\text{-}Mg}$
Perpendicular 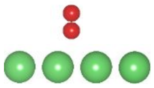	top	TUH1+fcc	-8.31	2.742	1.990, 1.989, 1.922, 1.993; 1.884, 1.949, 1.945
	bri	hcp+oct1	-8.41	2.839	1.903, 1.924, 1.924; 2.190, 2.142, 2.141, 2.249, 2.161, 2.260
	hcp	TUH1+fcc	-8.31	2.740	1.924, 1.992, 1.992, 1.994; 1.949, 1.884, 1.946
	fcc	fcc+oct1	-7.88	2.769	1.938, 1.941, 1.908; 1.997, 2.079, 2.080, 2.219, 2.211, 2.229
Horizontal 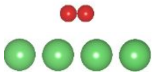	top-fcc	hcp+TUH1	-8.90	3.243	1.918, 1.908, 1.921; 1.960, 1.968, 1.954, 2.096
	top-hcp	TUH1+fcc	-8.31	2.740	1.992, 1.922, 1.991, 1.993; 1.945, 1.949, 1.885
	top-bri	oct1+fcc	-8.09	3.146	2.061, 1.964, 1.961, 2.127, 2.252, 2.236; 1.915, 1.925, 1.913
	fcc-top	hcp+oct1	-8.41	2.841	1.926, 1.903, 1.922; 2.253, 2.259, 2.160, 2.144, 2.189, 2.148
	hcp-top	hcp+oct1	-8.41	2.845	1.924, 1.904, 1.923; 2.147, 2.185, 2.146, 2.259, 2.183, 2.262
	bri-top	hcp+TUH1	-8.90	3.243	1.909, 1.918, 1.921; 1.970, 1.963, 1.959, 2.097
	bri-hcp	TUH1+TUH1	-9.07	3.141	1.970, 1.949, 1.963, 2.110; 1.950, 1.971, 1.964, 2.103

Table S3. Adsorption sites, adsorption energy (E_{ad}, eV), average adsorption energy ($E_{ad/av,}$ eV), stepwise adsorption energy (ΔE_{ad}, eV) under different coverages (ML) and two layers (32 O atoms), three layers (48 O atoms) and full oxidized (96 O atoms) Mg(0001) surfaces.

Number of O	Coverage (ML)	Site	E_{ad}	$E_{ad/av}$	ΔE_{ad}
1	0.0625	1×TUH1	-4.45	-4.45	-
2	0.125	2×TUH1	-9.08	-4.54	-4.62
2	0.125	Remote 2×TUH1	-8.89	-4.45	-
3	0.1875	3×TUH1	-13.82	-4.61	-4.74
4	0.25	3×TUH1; 1×TUH2	-18.52	-4.63	-4.71
4	0.25	4×TUH1	-18.39	-4.60	-
5	0.3125	4×TUH1; 1×TUH2	-23.46	-4.69	-4.93
5	0.3125	5×TUH1	-23.13	-4.63	-
6	0.375	4×TUH1; 2×TUH2	-28.27	-4.71	-4.82
6	0.375	6×TUH1;	-27.97	-4.66	-
7	0.4375	5×TUH1; 2×TUH2	-33.20	-4.74	-4.93
7	0.4375	6×TUH1; 1×hcp	-32.84	-4.69	-
8	0.5	5×TUH1; 3×TUH2	-38.05	-4.76	-4.85
8	0.5	6×TUH1; 2×TUH2	-37.90	-4.74	
9	0.5625	6×TUH1; 3×TUH2	-42.94	-4.77	-4.89
10	0.625	7×TUH1; 3×TUH2	-47.63	-4.76	-4.69
11	0.6875	7×TUH1; 4×TUH2	-52.45	-4.77	-4.82
12	0.75	8×TUH1; 4×TUH2	-57.39	-4.78	-4.94
13	0.8125	8×TUH1; 5×TUH2	-62.32	-4.79	-4.93
14	0.875	9×TUH1; 5×TUH2	-67.41	-4.81	-5.09
15	0.9375	9×TUH1; 6×TUH2	-72.31	-4.82	-4.91
16	1	10×TUH1; 6×TUH2	-77.16	-4.82	-4.85
32	2	16×TUH1; 16×TUH2	-158.70	-4.93	-
48	3	16×TUH1; 6×TUH2; 16×TUH3	-244.80	-5.10	-
96	6	96×TUH	-496.72	-5.17	-

Table S4. Adsorption energy ($E_{ad} = E_{2O/32O\_slab} - E_{32O\_slab} - E_{O2}$) and corresponding parameters for one O_2 molecule dissociation on the top two-layer fully oxidized Mg(0001) surface (32O).

**Initial site**		**Final site**	E_{ad}	$\mathbf{d_{O\text{-}O}}$	$\mathbf{d_{O\text{-}Mg}}$
Perpendicular	top	top*	-	1.34	1.88
	bri	fcc+fcc	-8.08	3.348	1.934, 1.927, 2.026, 1.935, 1.927, 2.025
	hcp	bri+bri	-4.53	1.563	2.007, 2.059, 2.007, 2.057,
	fcc	bri+bri	-4.53	1.560	2.007, 2.053, 2.008, 2.057
Horizontal	top	fcc+fcc	-8.08	3.348	1.934, 1.929, 2.024, 1.934, 1.929, 2.024
	hcp	bri+bri	-4.53	1.563	2.007, 2.057, 2.007, 2.057
	bri	bri+bri	-4.53	1.562	2.000, 2.058, 2.015, 2.055
	fcc	bri+bri-2	-4.10	1.539	1.999, 2.129, 2.004, 2.054

*denotes that the indicated site is not stable.

References

[1] Ismail, P. Hofmann, A.P. Baddorf, E.W. Plummer, Thermal expansion at a metal surface: A study of Mg(0001) and Be(10-10), Phys. Rev. B 66(24) (2002) 245414.
